# Clinical Laboratory Parameter–Driven Machine Learning for Participant Selection in Bioequivalence Studies Among Patients With Gastric Cancer: Framework Development and Validation Study

**DOI:** 10.2196/64845

**Published:** 2025-05-05

**Authors:** Byungeun Shon, Sook Jin Seong, Eun Jung Choi, Mi-Ri Gwon, Hae Won Lee, Jaechan Park, Ho-Young Chung, Sungmoon Jeong, Young-Ran Yoon

**Affiliations:** 1 Department of Medical Informatics, School of Medicine, Kyungpook National University, Daegu, Republic of Korea; 2 Center for Convergence Medical Research, School of Medicine, Kyungpook National University, Daegu, Republic of Korea; 3 Department of Molecular Medicine, School of Medicine, Kyungpook National University, 680 Gukchaebosang-ro, Jung-gu, Daegu, 41944, Republic of Korea, 82 534204950; 4 Department of Neurosurgery, School of Medicine, Kyungpook National University, Daegu, Republic of Korea

**Keywords:** machine learning, participant enrollment, clinical trial, eligibility criteria, clinical laboratory test, ML, support, electronic medical record, patient enrollment, model development, Korea, gastric cancer, framework, AI, artificial intelligence, trial

## Abstract

**Background:**

Insufficient participant enrollment is a major factor responsible for clinical trial failure.

**Objective:**

We formulated a machine learning (ML)–based framework using clinical laboratory parameters to identify participants eligible for enrollment in a bioequivalence study.

**Methods:**

We acquired records of 11,592 patients with gastric cancer from the electronic medical records of Kyungpook National University Hospital in Korea. The ML model was developed using 8 clinical laboratory parameters, including complete blood count and liver and kidney function tests, along with the dates of acquisition. Two datasets were collected: (1) a training dataset to design an ML-based candidate selection method and (2) a test dataset to evaluate the performance of the proposed method. The generalization performance of the ML-based method was confirmed using the *F*_1_-score and the area under the curve (AUC). The proposed model was compared with a random selection method to evaluate its efficacy in recruiting participants.

**Results:**

The weighted ensemble model achieved strong performance with an *F*_1_-score above 0.8 and an AUC value exceeding 0.8, demonstrating its ability to accurately identify valid clinical trial candidates while minimizing misclassification. Its high sensitivity further enhanced the model’s efficiency in prioritizing patients for screening. In a case study, the proposed ML model reduced the workload by 57%, efficiently identifying 150 valid patients from a pool of 209, compared to the 485 patients required by random selection.

**Conclusions:**

The proposed ML-based framework using clinical laboratory parameters can be used to identify patients eligible for a clinical trial, enabling faster participant enrollment.

## Introduction

Inadequate participant recruitment may lead to failure in a clinical trial, ultimately delaying new drug development and increasing costs [1-7]. Tasks comprising the recruitment process, such as prescreening, consent, screening, and communication between the participant and the study staff for the clinical trial, are inevitably labor intensive [2,3,8-10]. For instance, researchers manually review large volumes of electronic medical records (EMRs) during the prescreening process to identify potential candidates. Despite advances in the methodologies for each process in clinical trials, a systematic approach for enhancing the efficacy of participant enrollment is lacking.

Artificial intelligence (AI) has various applications in every section of the industry, and a machine learning (ML)–based optimal design of clinical trials has entered the sphere of pharmaceuticals [11]. Several AI techniques for identifying, screening, and enrolling appropriate participants for clinical trials have been introduced, and some have been employed commercially [1,4,8].

Corporates such as Mendel, Deep 6 AI, and Antidote have developed and provided AI solutions for clinical trial recruitment [12-16]. These commercial services use massive data such as demographics, laboratory, imaging, and multiomics data to facilitate faster recruitment and provide full-service recruitment [14,15]. Jin et al [17] reported that a result from a user study using large language model framework, TrialGPT, to support patient matching resulted in a 42.6% decrease in the screening time [18]. Another AI approach is clinical trial digital twin technology [18-21]. Digital twin technology creates virtual patients that replicate individual characteristics, enabling the prediction of clinical responses [18,19,21]. By utilizing digital twins, the required sample sizes for clinical trials can be reduced [18,19,21]. These informatics-based tools are expected to improve recruitment efficiency, directly influencing the success rate of clinical trials [1,4,5].

Eligibility criteria specify the qualification of participants in clinical trials, and this component comprises structured information, including diagnoses and laboratory results, and unstructured information, such as clinical free text. Previous AI studies have primarily focused on developing advanced natural language processing and optical character recognition techniques for extracting unstructured data [2,3,6,8-10]. As AI technology is rapidly changing, most research and services have developed AI tools using both structured and unstructured data [12,14-16,22]. However, complicated preprocesses (eg, annotation), discrepancies between EMR systems and databases of institutions (eg, noninteroperable algorithms), and high costs limit their application. Moreover, highly sophisticated eligibility criteria can render the generalization of algorithms challenging.

In contrast, clinical laboratory test data and typical structured data can be readily incorporated into an EMR-utilizing algorithm, given that these data are comparatively more objective than other unstructured data [23,24]. Mohammad et al [11] have suggested that disclosing the pretest probability of a specific test result can be clinically meaningful for some laboratory tests.

In bioequivalence studies of drugs, clinical laboratory tests such as hematology and blood chemistry tests are included as eligibility criteria. Participants considered suitable by the investigator based on laboratory results are allowed to take part in the bioequivalence study. Therefore, prescreening through clinical laboratory tests is considered to be effective in quickly selecting potential candidates.

Accordingly, we formulated an ML-based framework to identify participants using the clinical laboratory test values of candidates. In this study, we chose to compare the ML-based method with a random selection method, which we considered representative of the common practice in clinical settings where patient lists are screened sequentially. Collectively, the objective of this study was to develop a simple and rapidly applicable ML algorithm that could assist in participant identification for bioequivalence study enrollment.

## Methods

### Study Design

In total, records of 11,592 patients with gastric cancer were acquired from the EMRs of Kyungpook National University Hospital in Korea from 2011 to 2019. Clinical laboratory parameters (including complete blood count and liver and kidney function tests) and acquisition dates were acquired to develop the ML-based model to predict relevant laboratory data of patients on a future date. The laboratory parameters selected were those used in the eligibility criteria for bioequivalence testing of anticancer drugs in patients with gastric cancer. We developed the model using 8 basic and straightforward parameters such as complete blood count (hemoglobin, neutrophil count, platelet count), liver function tests (total bilirubin, aspartate aminotransferase, alanine aminotransferase, alkaline phosphatase), and kidney function tests (creatinine), which are minimal tests capable of assessing health status. These parameters enable a simple and efficient preliminary assessment of potential screening candidates.

“Label 1” was assigned when the candidate’s clinical laboratory data fell within the valid range, while “label 0” indicated data outside the valid range (Table 1). A patient was considered a valid candidate for a clinical trial only if all predicted laboratory data met the eligibility criteria. To design an ML-based candidate selection method, the dataset was divided into training and test sets; data collected from 2011 to 2018 were used for training, while data from 2019 were reserved for testing (Table 1). This time-based split ensured a fair evaluation of the model’s performance by avoiding any temporal overlap, thereby reflecting real-world scenarios where future data (test data) would not be available during model training. Additionally, this approach allowed us to assess the model’s ability to generalize unseen data while accounting for potential temporal effects, such as advances in medical technology or shifts in population health trends. By adopting this methodology, we aimed to provide a realistic evaluation of the model’s performance in clinical settings.

**Table 1.** Distribution of training and test sets based on the validity of parameters.

Laboratory parameters	Valid range (label 1)	Training data points (2011 to 2018), n			Test data points (2019), n		
		Total	Label 0	Label 1	Total	Label 0	Label 1
Hemoglobin	13-18 g/dL	92,790	18,259	74,531	11,664	1652	10,012
Neutrophil count	40-74 %	68,622	39,210	29,412	7703	4212	3491
Platelet count	130-400 1000/µL	83,031	63,435	19,596	10,025	7258	2767
Bilirubin	0-1.2 mg/dL	68,313	56,017	12,296	8921	7060	1861
AST^a^	0-40 U/L	9235	7405	1830	964	710	254
ALT^b^	40-41 U/L	9157	7704	1453	961	768	193
ALP^c^	40-129 U/L	8712	7020	1692	884	737	147
Creatinine	0.7-1.2 mg/dL	69,467	40,053	29,414	8890	3525	5365

a AST: aspartate aminotransferase.

b ALT: alanine aminotransferase.

c ALP: alkaline phosphatase.

### Ethical Considerations

This study was approved by the Kyungpook National University Hospital Institutional Review Board (KNUH 2020-04-023), and a waiver of consent was authorized. All research was performed in accordance with the guidelines of the Declaration of Helsinki, and only deidentified data were used and analyzed in our retrospective study.

### Data Preprocessing

We attempted to resolve the issues of aperiodicity and imbalance in the training dataset by applying the combination method and principal component analysis (PCA). First, to analyze the trend of laboratory data changes, at least 3 sequential data points of laboratory data and acquisition dates were required. However, given the nonperiodical patient visits to the hospital, the intervals between each data point were inconsistent. To compensate for the aperiodicity of the data, the distribution of data points was increased by computing a simple combination method as follows:

$C(n,k)=\frac{P\left(n,\;k\right)}{k!}=\frac{n!}{\left(n-k\right)!k!}$ (1)

where *P* indicates the permutation function, *n* is the number of data points, and *k* is the number of selected sequential data points (4 in this study). Therefore, 4 sequential data points with 8 values, comprising 6 initial values to analyze the trend of data changes and 2 values as target data to verify the analysis model, were generated as one data group. For example, if a patient had 10 laboratory data points, 210 data groups, including 8 values, were augmented. Each laboratory data value was normalized by the minimum and maximum values of data distribution, and the acquisition date values are represented by |acquisition date − a screening date| / *N*, where *N* is a normalization factor. Finally, each data group was encoded by considering whether the last 2 values were within the valid range. Table S1 in Multimedia Appendix 1 presents the number of training data points obtained using the combination method.

**Figure 1.**
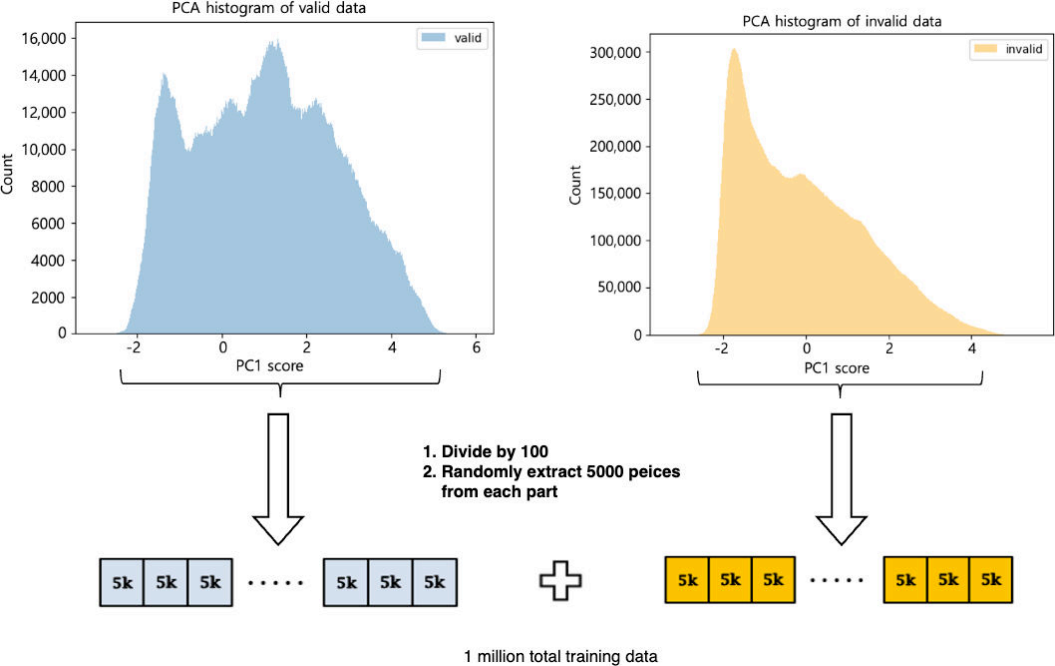
Training dataset selection procedure. PCA: principal component analysis; PC1: first principal component.

The augmented training dataset was intuitively visualized by applying the first principal component, as shown in Figure 1. Although Equation 1 helps resolve aperiodicity, the problem of nonuniform distribution in the dataset persisted in both valid and invalid classes. Thus, to compensate for the nonuniform distribution of training data groups, we normalized the imbalanced data into 100,000 even training data points of each laboratory parameter by dividing the data distribution into 100 regions and randomly extracting 5000 data points from each region (Figure 1). Moreover, we attempted to correct the data imbalance by selecting the same number of data points for each label class. Table S2 in Multimedia Appendix 1 lists the numbers of training and test datasets.

### ML-Based Selection Method

There are various models in ML, and the performance of each model can vary greatly depending on how the elements of the process, such as data preprocessing, feature engineering, model selection, hyperparameter tuning, and ensembling, are configured. Therefore, specialized knowledge and experience are required, making it difficult for nonexperts to use the same skills as experts. To solve this problem, automated ML (AutoML) simplifies and automates the mentioned complex processes, so that nonexperts can easily use ML and expect the same performance as experts.

**Figure 2.**
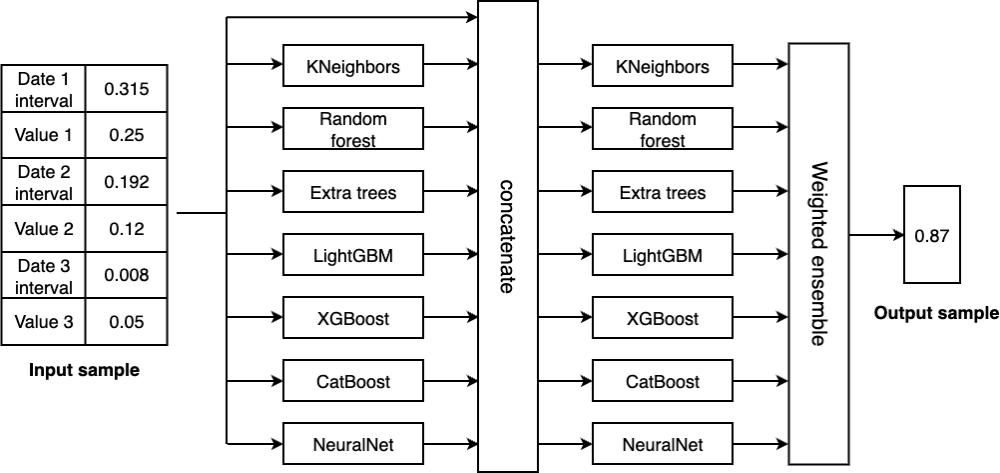
The best model architecture designed by AutoGluon-Tabular. LightGBM: Light Gradient-Boosting Machine; XGBoost: Extreme Gradient Boosting.

AutoGluon-Tabular is an AutoML and an open-source library developed by Amazon Web Services [25]. Figure 2 shows the architecture in this study that utilizes it. The input data is applied with various ML models (KNeighbors, random forest, Extra Trees, Light Gradient-Boosting Machine [LightGBM], Extreme Gradient Boosting [XGBoost], CatBoost, and NeuralNet) and a stacked ensemble technology that combines them, and the output is the probability value that predicts whether the patient is valid.

## Results

The present study consisted of two parts: (1) the generalization performance of the ML-based model was confirmed with various evaluation metrics, including accuracy, sensitivity, specificity, precision, *F*_1_-score, and area under the curve (AUC); and (2) the proposed model was compared using a random selection method to evaluate its efficacy in participant recruitment.

The training results for each model were evaluated as *F*_1_-scores (Table 2). The weighted ensemble model resulted in the highest average *F*_1_-score of 0.91 (SD 0.076). The weighted ensemble model exhibited the best performance in terms of precision and recall. Among the same ML models, detailed models were classified according to parameters such as the size and calculation method. As shown in Table 2, we observed that the *F*_1_-score for the weighted ensemble model was over 0.8 during the training process, indicating strong performance. An *F*_1_-score above 0.8 is generally considered strong, as it reflects a good balance between precision and recall, which is crucial in clinical applications where both false positives and false negatives can have significant consequences. Precision measures how many of the predicted valid candidates are truly valid, while recall reflects how many of the actual valid candidates were correctly identified by the model. A high *F*_1_-score ensures that the model is not only accurate in predicting valid candidates but also minimizes the risk of misclassifying candidates who could otherwise be eligible for clinical trials.

We evaluated the performance of the classification task of the ML algorithm (Table 3). The receiver operating characteristic curves for each clinical parameter are shown in Figure S1 in Multimedia Appendix 1. In Table 3, the overall AUC value exceeded 0.8, demonstrating high performance. An AUC value exceeding 0.8 is considered high, indicating that the model has a strong ability to discriminate between valid and invalid candidates. The AUC measures the model’s ability to distinguish between eligible and noneligible patients, and an AUC value closer to 1 signifies excellent performance. This is particularly important in clinical settings, where the model’s ability to prioritize the right candidates can improve the efficiency and accuracy of the screening process. What we found particularly noteworthy in the results shown in Table 3 was the high sensitivity. A higher sensitivity means that the probability of correctly identifying patients who fall within the valid range is increased. We can prioritize patients with a higher probability of being in the valid range based on model predictions, and by sequentially conducting additional tests, quickly screen for an appropriate and statistically significant sample size for clinical trials, ultimately contributing to a more efficient and successful trial.

For the application test, we evaluated the effectiveness of the proposed model for identifying eligible patients from the test dataset. First, assuming prescreening dates from December 1, 2019, to December 31, 2019, laboratory data were collected from 2019. One test dataset was constructed by retrieving the last 3 laboratory data points per patient during the period. Table 4 presents the number of clinical trial participants used for the application test. In the actual hospital EMR, no alkaline phosphatase test data were available for patients with gastric cancer during this period. Cases were classified according to their valid ratio to the total. We designated the parameters as case 1 when the number of valid data was <50%, case 2 when it ranged between 50% and 60%, and case 3 when it was >60%.

**Figure 3.**
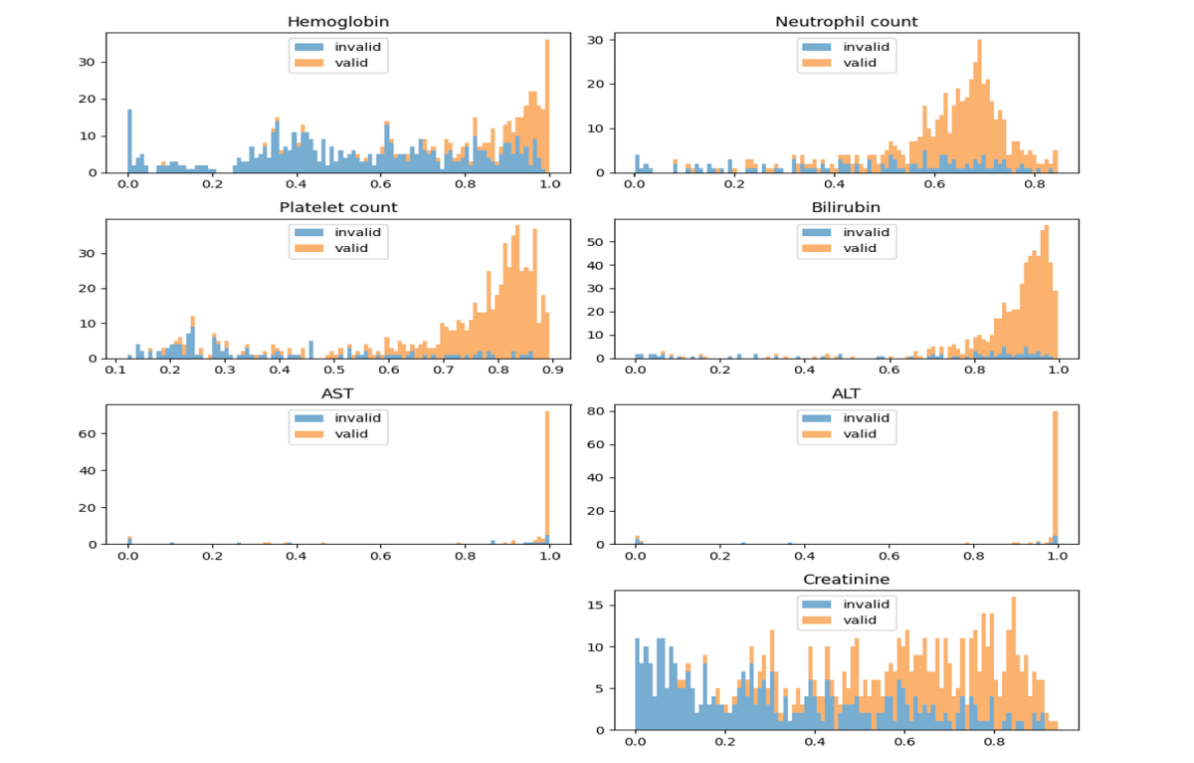
Probability histogram stacked with invalid and valid number of patients (x-axis: probability predicted to be valid; y-axis: number of patients). ALT: alanine aminotransferase; AST: aspartate aminotransferase.

Figure 3 shows the number of valid and invalid patients by probability from 0 to 1. The number of invalid patients by probability was first drawn as a histogram, and then the valid patients were drawn on top of it by accumulating them. As can be seen in the results, there were fewer valid patients and more invalid patients on the side where the predicted probability was close to 0, while there were more valid patients and fewer invalid patients on the side where the probability was close to 1. Through this, we can see that the results predicted by ML are reliable, and it is advantageous to select patients with high probability values for patient screening.

**Figure 4.**
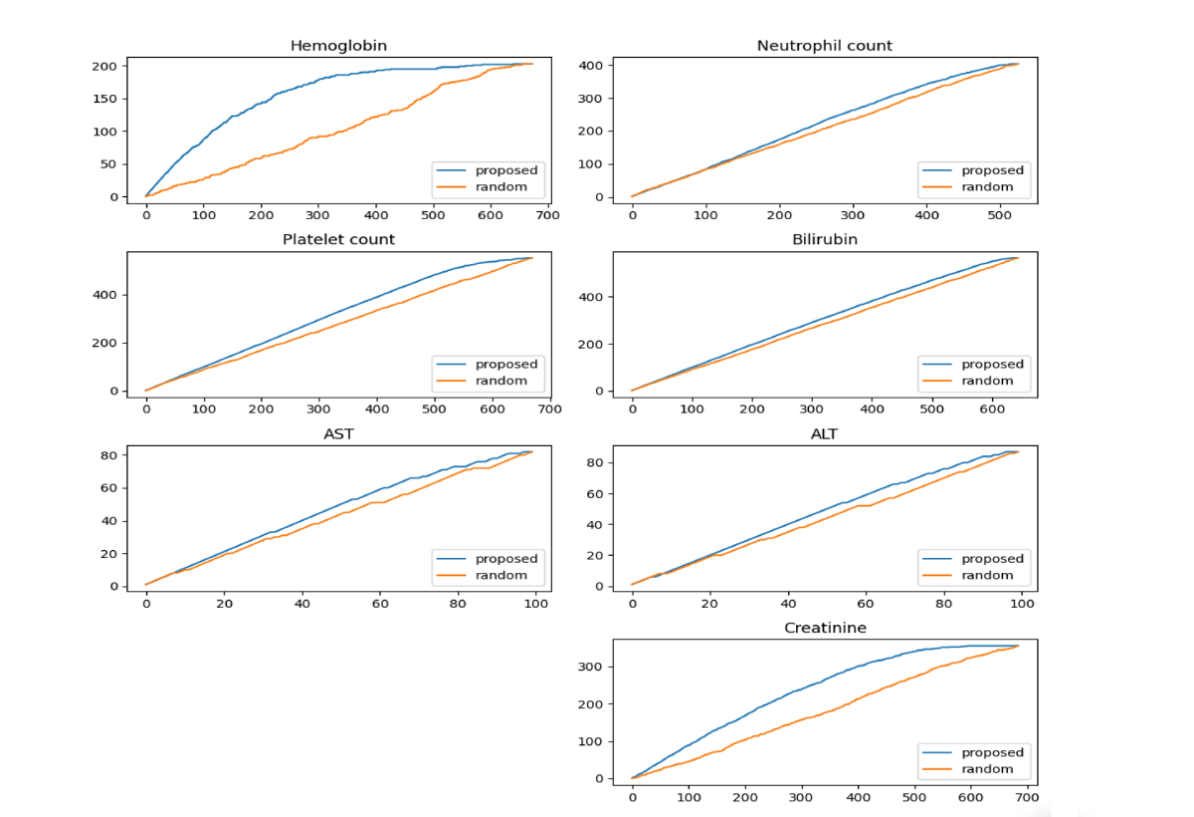
Accumulative results of valid participant identification for clinical trials (x-axis: number of predicted data; y-axis: number of valid data). ALT: alanine aminotransferase; AST: aspartate aminotransferase.

Finally, valid candidates were identified by extracting patients in the order of highest predicted probability from the proposed model, and the results were compared with random findings. Figure 4 shows the results concerning the identification of valid patients for clinical trials. For example, in the case of hemoglobin, we aimed to identify 150 clinically suitable patients out of 673 total patients, where 203 patients met the eligibility criteria. As shown in Figure 4, our proposed model identified 150 valid patients by screening only 209 high-probability candidates. In contrast, a random selection process required screening 485 patients to find 150 valid patients. This demonstrates an approximate 57% reduction in workload.

**Figure 5.**
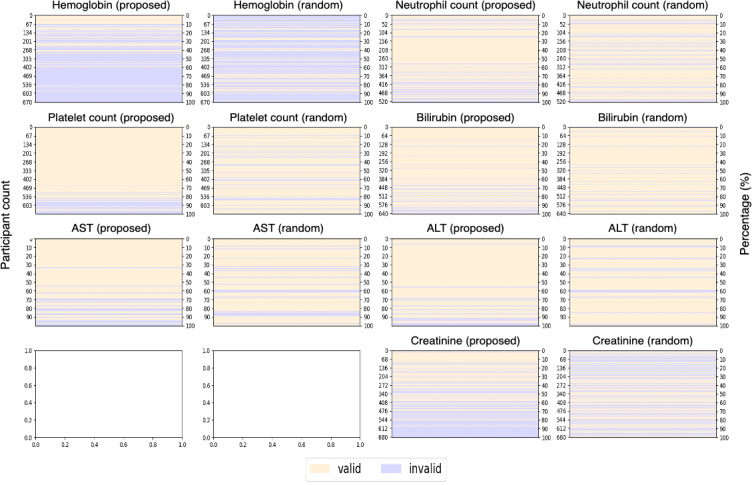
Comparison of clinical trial participants in the order of probability of the proposed model (proposed) and random (random). ALT: alanine aminotransferase; AST: aspartate aminotransferase.

Figure 5 shows a comparison of clinical trial participants in the order of probability of the proposed model and random method. Compared with the random selection method, more valid patients were distributed at the top of the proposed result, and more invalid patients were distributed at the bottom. When selecting patients for clinical trials, if the patients sorted by the proposed method are assigned from the top, it is possible to determine suitable target patients faster than with the random method.

Overall, the proposed ML model identified valid participants for clinical trials faster than the random selection method. In particular, as seen in the case of hemoglobin and creatinine, in case 1 and case 2, that is, when the valid rate was <60%, the proposed model identified valid participants considerably faster.

**Table 2.** The training results of the classification task evaluated by the *F*_1_-score.

Model	*F*_1_-scores							
	Hemoglobin	Neutrophil count	Platelet count	Bilirubin	AST^a^	ALT^b^	ALP^c^	Creatinine
Weighted ensemble	0.8816	0.7980	0.8537	0.9011	0.9894	0.9874	0.9943	0.8412
LightGBM^d^	0.8475	0.7790	0.7309	0.8724	0.9814	0.9823	0.9912	0.7589
LightGBM Large	0.8755	0.6695	0.7482	0.8980	0.9894	0.9874	0.9943	0.7642
LightGBM XT	0.7809	0.6559	0.7184	0.7483	0.9088	0.7931	0.9361	0.7535
RandomForest Gini	0.8680	0.7866	0.8524	0.8740	0.9776	0.9784	0.9891	0.8372
RandomForest Entr	0.8708	0.7861	0.8525	0.8735	0.9783	0.9780	0.9888	0.8397
ExtraTrees Gini	0.8684	0.7808	0.8472	0.8719	0.9765	0.9776	0.9853	0.8327
ExtraTrees Entr	0.8686	0.7791	0.8461	0.8695	0.9776	0.9777	0.9849	0.8318
XGBoost^e^	0.7610	0.6776	0.7281	0.7920	0.9254	0.9099	0.9476	0.7628
CatBoost	0.6941	0.6479	0.7214	0.7517	0.8266	0.8548	0.8875	0.7547
NeuralNet MXNet	0.7750	0.6883	0.7715	0.7867	0.9024	0.9161	0.9245	0.7778
NeuralNet FastAI	0.7435	0.6842	0.7493	0.7621	0.8373	0.8167	0.8791	0.6969
KNeighbors Dist	0.7973	0.6704	0.7692	0.6932	0.9030	0.9116	0.7530	0.5864
KNeighbors Unif	0.7906	0.6612	0.7639	0.6921	0.8880	0.8942	0.7512	0.5928

a AST: aspartate aminotransferase.

b ALT: alanine aminotransferase.

c ALP: alkaline phosphatase.

d LightGBM: Light Gradient-Boosting Machine.

e XGBoost: Extreme Gradient Boosting.

**Table 3.** Performance test results.

Clinical parameter	Accuracy	Sensitivity	Specificity	Precision	*F*_1_-score	AUC^a^
Hemoglobin	0.664	0.950	0.617	0.290	0.445	0.915
Neutrophil count	0.756	0.909	0.573	0.719	0.803	0.797
Platelet count	0.851	0.906	0.706	0.890	0.898	0.893
Bilirubin	0.847	0.915	0.588	0.894	0.904	0.826
AST^b^	0.767	0.832	0.583	0.848	0.840	0.789
ALT^c^	0.850	0.887	0.705	0.923	0.904	0.864
ALP^d^	0.889	0.954	0.565	0.917	0.935	0.836
Creatinine	0.792	0.818	0.775	0.705	0.757	0.867

a AUC: area under the curve.

b AST: aspartate aminotransferase.

c ALT: alanine aminotransferase.

d ALP: alkaline phosphatase.

**Table 4.** Number of clinical trial participants for application test.

Clinical parameters	Test set for application test				
	Total participants, n	Valid participants, n	Invalid participants, n	Valid rate (%)	Case number
Hemoglobin	673	203	470	30.16	1
Neutrophil count	525	404	121	76.95	3
Platelet count	670	553	117	82.54	3
Bilirubin	644	566	78	87.89	3
AST^a^	100	82	18	82	3
ALT^b^	100	87	13	87	3
ALP^c^	0	0	0	—^d^	—
Creatinine	684	356	328	52.05	2

a AST: aspartate aminotransferase.

b ALT: alanine aminotransferase.

c ALP: alkaline phosphatase.

d Not available.

## Discussion

### Principal Findings

We developed an ML-based framework to support the prescreening of clinical research using clinical laboratory tests to reduce the workload of reviewing structured data of eligibility criteria. We developed a protypical but practical framework using clinical laboratory data and achieved high accuracy and a 57% workload reduction compared to random selection when identifying 150 valid patients. Based on our results, we expect that the framework will potentially reduce investigators’ burden and shorten the recruitment period by assisting with conventional manual prescreening.

Prescreening involves the process of selecting candidates for screening, a cumbersome manual task that requires a heavy burden of research personnel at this stage [2,3,8-10]. The most efficient known method for determining eligibility criteria–matching candidates is as follows: candidates are randomly prioritized by research staff, and one staff member is selected to review and narrow the potential list for screening. Although several AI-based tools for assisting the recruitment of participants have been introduced, there are a few tools to support prescreening [1], and relevant studies using only structured information, such as laboratory results, are limited. Recently developed AI-based recruitment services offer a significant advantage in performing precise and rapid clinical trial participant matching by incorporating both structured and unstructured data. However, these services can be costly to implement. In contrast, our study suggests a practical alternative to more comprehensive but potentially cost-prohibitive AI-based recruitment services by showing that efficient prescreening can be conducted using laboratory data alone. This approach provides a more accessible and cost-effective method for individual researchers to apply in their bioequivalence studies.

Considering the framework of this study, the primary contribution is the establishment of a ranked list of potential participants for trial enrollment in an intuitive manner. A simple list of potential candidates can be valuable in informing the decision to review additional eligibility criteria, thereby alleviating the burden of manual prescreening and preventing underestimation due to manual review. Advantages of the framework include simple and time-saving characteristics, the requirement for only a few laboratory data among numerous EMR data, and effortless implementation through AutoGluon-Tabular. The utilization of programs such as AutoGluon-Tabular makes it easy for beginners to employ ML. In addition, ML with techniques tuned for performance improvement can be utilized by experts with minimal time and effort, making our approach more accessible.

We speculate that the irregularity of patient visiting schedules and the imbalance of the clinical laboratory parameters remain major driving limitations in the present ML-based framework. To overcome these defects, we carefully designed the algorithm using the PCA method (Table S2 in Multimedia Appendix 1 and Figure 1). However, further research is required to refine the solutions to combat aperiodicity and imbalanced data.

The proposed system can be integrated into clinical trial recruitment workflows as a decision-support tool for the prescreening process. Currently, prescreening candidates for clinical trials often involves manually reviewing large volumes of patient data to determine eligibility. By automating the selection process, our system can generate a ranked list of potential participants based on clinical laboratory parameters, allowing research staff to focus their manual review efforts on high-priority candidates. This integration could be achieved by embedding the system into existing EMR systems. The framework can process patient data directly from the EMR, analyze the eligibility criteria, and provide a prioritized candidate list in real time. Additionally, the ranked list can be updated dynamically as new patient data becomes available, ensuring that the recruitment process is both efficient and adaptive to changing conditions.

The clinical data used in this study were collected from a specific institution (Kyungpook National University Hospital) and from patients with a specific condition, which may limit the generalizability of the findings. Therefore, further diversification of the data and additional validation are necessary. Moreover, in terms of ethics, AI models should not replace human judgment in the clinical trial participant selection process, but rather serve as an assistive tool for initial screening. In addition to the limitations of generalizability due to the data source, integrating this system into clinical workflows may encounter challenges such as compatibility with diverse EMR platforms, requiring standardized data formats and robust interoperability. Training research staff to effectively use the system and ensuring transparency in AI decision-making processes will be critical for fostering trust and adoption. Moreover, ethical and regulatory compliance regarding patient data usage must be carefully managed to address privacy concerns and uphold clinical trial standards

Our future work will focus on improving the generalization of the framework. First, we plan to apply this method to screen patients for bioequivalence studies involving cancers other than gastric cancer. Bioequivalence studies of anticancer drugs are mostly conducted on patients under follow-up after curative treatment and are performed to demonstrate the equivalence of bioavailability. Therefore, compared to clinical trials aimed at evaluating efficacy, the eligibility criteria are not quite complicated. Additionally, the presence of similar criteria makes the framework developed in this study likely to be easily generalizable.

Second, while researchers typically utilize both structured and unstructured data in the screening process, this study is limited to the use of structured data. However, we expect that screening with an ML-based framework, followed by incorporating unstructured data, could help reduce the overall workload. We aim to enhance the framework to integrate unstructured data for a more comprehensive screening process. Third, we recognize the importance of comparing our approach to existing methods, and plan to include such comparisons in future research. In this study, we targeted structured information from patients; considering unstructured and structured information may therefore enhance the generalization of the framework, covering comprehensive eligibility criteria. In addition, we plan to conduct a prospective study to compare the proposed framework with conventional methods. Collectively, we anticipate that the framework will cover a wide disease spectrum and expand its applicability to clinical trials from other institutions.

### Conclusion

We proposed an AI support framework utilizing structured information on eligibility criteria to select appropriate candidates for clinical trial enrollment. This method could accelerate the efficiency of prescreening processes and can be applied to various clinical trials.

## Supplementary material

10.2196/64845 Multimedia Appendix 1 Supplementary materials regarding the number of training data points obtained using the combination method, the training and test datasets, and receiver operating characteristic curves of performance test results.

## Abbreviations

AI: artificial intelligence
AUC: area under the curve
AutoML: automated machine learning
EMR: electronic medical record
LightGBM: Light Gradient-Boosting Machine
ML: machine learning
PCA: principal component analysis
XGBoost: Extreme Gradient Boosting

## References

[1] Jain NM, Culley A, Knoop T, Micheel C, Osterman T, Levy M (2019). Conceptual framework to support clinical trial optimization and end-to-end enrollment workflow. JCO Clin Cancer Inform.

[2] Meystre SM, Heider PM, Kim Y, Aruch DB, Britten CD (2019). Automatic trial eligibility surveillance based on unstructured clinical data. Int J Med Inform.

[3] Ni Y, Kennebeck S, Dexheimer JW (2015). Automated clinical trial eligibility prescreening: increasing the efficiency of patient identification for clinical trials in the emergency department. J Am Med Inform Assoc.

[4] Harrer S, Shah P, Antony B, Hu J (2019). Artificial intelligence for clinical trial design. Trends Pharmacol Sci.

[5] Zhavoronkov A, Vanhaelen Q, Oprea TI (2020). Will artificial intelligence for drug discovery impact clinical pharmacology?. Clin Pharmacol Ther.

[6] Majeed RW, Röhrig R (2011). Identifying patients for clinical trials using fuzzy ternary logic expressions on HL7 messages. Stud Health Technol Inform.

[7] Ledford H (2011). Translational research: 4 ways to fix the clinical trial. Nature.

[8] Ni Y, Wright J, Perentesis J (2015). Increasing the efficiency of trial-patient matching: automated clinical trial eligibility pre-screening for pediatric oncology patients. BMC Med Inform Decis Mak.

[9] (2016). The age of analytics: competing in a data-driven world. McKinsey & Company.

[10] Jeong E, Park N, Choi Y, Park RW, Yoon D (2018). Machine learning model combining features from algorithms with different analytical methodologies to detect laboratory-event-related adverse drug reaction signals. PLoS One.

[11] Mohammad F, Theisen-Toupal JC, Arnaout R (2014). Advantages and limitations of anticipating laboratory test results from regression- and tree-based rules derived from electronic health-record data. PLoS One.

[12] Ismail A, Al-Zoubi T, El Naqa I, Saeed H (2023). The role of artificial intelligence in hastening time to recruitment in clinical trials. BJR Open.

[13] Beck JT, Rammage M, Jackson GP (2020). Artificial intelligence tool for optimizing eligibility screening for clinical trials in a large community cancer center. JCO Clin Cancer Inform.

[14] Mendel for providers. Mendel.

[15] Deep 6 AI.

[16] Antidote.

[17] Jin Q, Wang Z, Floudas CS (2024). Matching patients to clinical trials with large language models. Nat Commun.

[18] Hutson M (2024). How AI is being used to accelerate clinical trials. Nature New Biol.

[19] Das T, Wang Z, Sun J (2023). KDD ’23: Proceedings of the 29th ACM SIGKDD Conference on Knowledge Discovery and Data Mining.

[20] Wang Y, Fu T, Xu Y (2024). TWIN-GPT: digital twins for clinical trials via large language model. ACM Trans Multimedia Comput Commun Appl.

[21] Zhang B, Zhang L, Chen Q, Jin Z, Liu S, Zhang S (2023). Harnessing artificial intelligence to improve clinical trial design. Commun Med (Lond).

[22] Askin S, Burkhalter D, Calado G, El Dakrouni S (2023). Artificial intelligence applied to clinical trials: opportunities and challenges. Health Technol (Berl).

[23] Haddad T, Helgeson JM, Pomerleau KE (2021). Accuracy of an artificial intelligence system for cancer clinical trial eligibility screening: retrospective pilot study. JMIR Med Inform.

[24] Zeng K, Xu Y, Lin G, Liang L, Hao T (2021). Automated classification of clinical trial eligibility criteria text based on ensemble learning and metric learning. BMC Med Inform Decis Mak.

[25] Erickson N, Mueller J, Shirkov A (2020). AutoGluon-Tabular: robust and accurate automl for structured data. arXiv.

