# Supplementary material for: Clinical Laboratory Parameter–Driven Machine Learning for Participant Selection in Bioequivalence Studies Among Patients With Gastric Cancer: Framework Development and Validation Study
Source: JMIR AI. 2025 May 5;4:e64845. doi: 10.2196/64845 (PMC12223687; doi:10.2196/64845)
Supplement: Multimedia Appendix 1 [file ai-v4-e64845-s001.docx]

Table S1. The number of data groups by the combination method

|  | **Training data groups (2011 to 2018)** | | |
| --- | --- | --- | --- |
|  | **Total** | **Label 0** | **Label 1** |
| Hemoglobin | 97,218,130 | 89,518,706 | 7,699,424 |
| Neutrophil count | 68,220,673 | 36,493,908 | 31,726,765 |
| Platelet count | 79,104,290 | 31,948,443 | 47,155,847 |
| Bilirubin | 42,023,683 | 10,574,287 | 31,449,396 |
| AST | 3,593,919 | 1,291,987 | 2,301,932 |
| ALT | 3,526,298 | 846,264 | 2,680,034 |
| ALP | 3,944,296 | 1,697,000 | 2,247,296 |
| Creatinine | 49,859,989 | 28,316,961 | 21,543,028 |

Abbreviations: AST, aspartate aminotransferase, ALT, alanine aminotransferase, ALP, alkaline phosphatase

Table S2. The number of train and test dataset after preprocessing

| **Types of lab data** | **Trainset** | | | **Testset for performance test** | | |
| --- | --- | --- | --- | --- | --- | --- |
|  | **Total** | **Valid** | **Invalid** | **Total** | **Valid** | **Invalid** |
| Hemoglobin | 1,000,000 | 500,000 | 500,000 | 11,664 | 1,652 | 10,012 |
| Neutrophil count | 1,000,000 | 500,000 | 500,000 | 7,703 | 4,212 | 3,491 |
| Platelet count | 1,000,000 | 500,000 | 500,000 | 10,025 | 7,258 | 2,767 |
| Bilirubin | 1,000,000 | 500,000 | 500,000 | 8,921 | 7,060 | 1,861 |
| AST | 1,000,000 | 500,000 | 500,000 | 964 | 710 | 254 |
| ALT | 1,000,000 | 500,000 | 500,000 | 961 | 768 | 193 |
| ALP | 1,000,000 | 500,000 | 500,000 | 884 | 737 | 147 |
| Creatinine | 1,000,000 | 500,000 | 500,000 | 8,890 | 3,525 | 5,365 |

Abbreviations: AST, aspartate aminotransferase, ALT, alanine aminotransferase, ALP, alkaline phosphatase

**
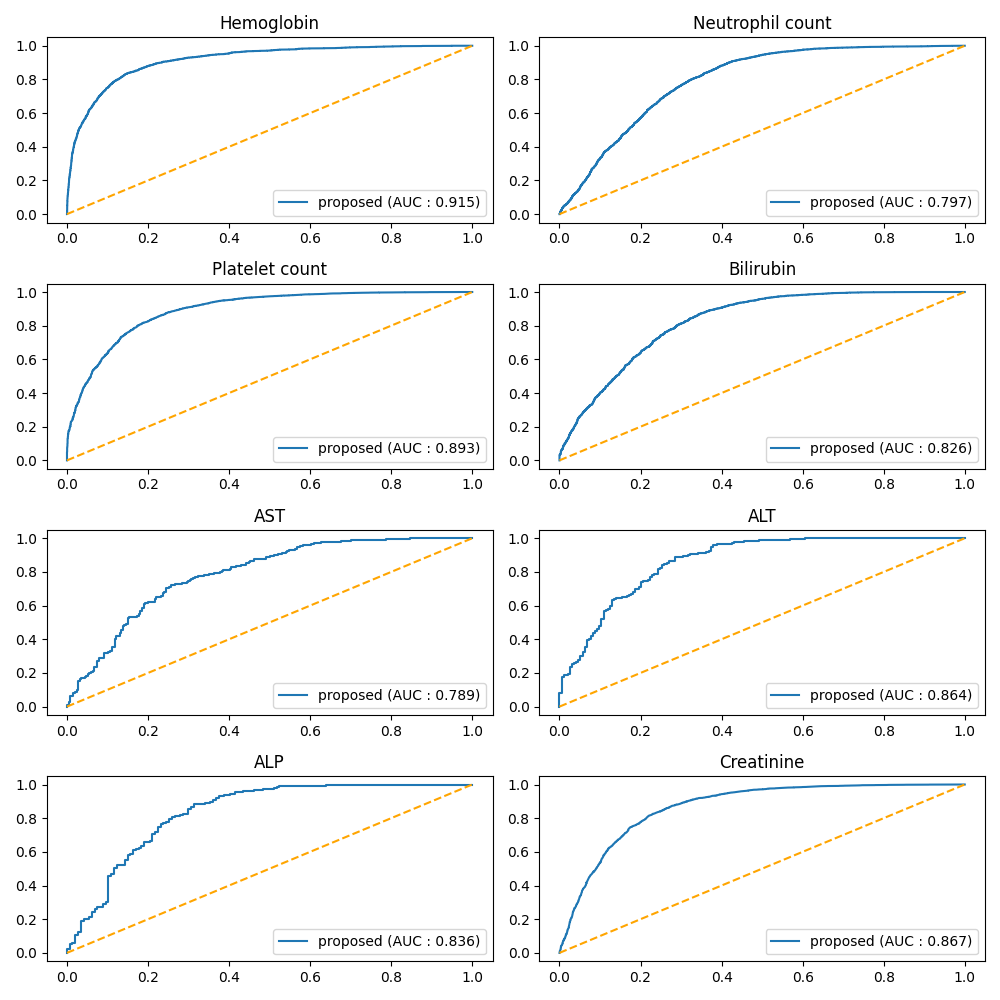
**

Figure S1. ROC curve of performance test results

( X : false positive rate, Y : true positive rate )
